# Anti-Inflammatory Activity of Iridoids and Verbascoside Isolated from *Castilleja tenuiflora*

**DOI:** 10.3390/molecules181012109

**Published:** 2013-09-30

**Authors:** Danae Carrillo-Ocampo, Sugeyla Bazaldúa-Gómez, Jaime R. Bonilla-Barbosa, Rola Aburto-Amar, Verónica Rodríguez-López

**Affiliations:** 1 Facultad de Farmacia, Universidad Autónoma del Estado de Morelos, Av. Universidad 1001, Col. Chamilpa., Cuernavaca 62209, Morelos, Mexico; E-Mails: cod_ff@uaem.mx (D.C.-O.); sugeylabazaldua@grupomedifarma.com (S.B.-G.); 2 Centro de Investigaciones Biológicas, Universidad Autónoma del Estado de Morelos, Av. Universidad 1001, Col. Chamilpa., Cuernavaca 62209, Morelos, Mexico; E-Mail: bonilla@cib.uaem.mx; 3 Instituto de Farmacobiología, Universidad de la Cañada, Carretera Teotitlán - San Antonio Nanahuatipán Km 1.7 s/n., Paraje Titlacuatitla, Teotitlán de Flores Magón 68540, Oax., Mexico; E-Mail: rola_amar@unca.edu.mx

**Keywords:** *Castilleja tenuiflora*, anti-inflammatory activity, iridoids, TPA

## Abstract

*Castilleja tenuiflora* (Orobanchaceae) has been used in Mexican traditional medicine as a treatment for cough, dysentery, anxiety, nausea and vomiting as well as hepatic and gastrointestinal diseases. The ethanolic extract of the aerial parts of *Castilleja tenuiflora* was separated by silica gel column chromatography. The fractions were evaluated using the induced edema acetate 12-*O*-tetradecanoylphorbol (TPA) anti-inflammatory activity model. The most active fraction was subjected to medium-pressure liquid chromatography (MPLC) with UV detection at 206 and 240 nm. The following iridoids were isolated: geniposidic acid, aucubin, bartioside, 8-*epi*-loganin, mussaenoside, and the phenylpropanoid verbascoside. The most active iridoid was geniposidic acid, which was more active than the control (indomethacin), and the least active iridoid was mussaenoside. 8-*epi*-Loganin, and mussaenoside have not been previously reported to be anti-inflammatory compounds. The results of these investigations confirm the potential of Mexican plants for the production of bioactive compounds and validate the ethnomedical use of *Castilleja tenuiflora*-like anti-inflammatory plants.

## 1. Introduction

The genus *Castilleja* comprises more than 220 species, which are mainly distributed in America. This genus was reclassified from the family Scrophulariaceae into the family Orobanchaceae based on molecular phylogenetic analyses [1]. *Castilleja tenuiflora* Benth. (synonyms: *C angustifolia* M. Martens & Galeotti and *C. canescens* Benth.) is an annual herb distributed in North America and is commonly known in Mexico as “garaiiona”, “cola de borrego” (lamb tail), or “hierba del cancer” [2]. It is a small perennial herb of 1 m in height and is found in disturbed areas of pine oak temperate forests. A decoction of the leaves and flowers is used for coughs and in the treatment of dysentery, nerves, nausea, and vomiting as well as hepatic and gastrointestinal diseases [1]. Previous studies have reported the presence of five acetylated glycoside iridoids. The following iridoids have been isolated: aucubin hexacetate, bartsioside pentacetate, and mixture of carboxylic acids, which were methylated afforded geniposidic acid methyl ester pentaacetate, mussaenosidic acid methyl ester tetraacetate and shanzhiside methyl ester pentaacetate [3]. Recently, verbascoside and isoverbascoside were isolated from the roots [1]. More extensive studies have revealed that iridoids exhibit a wide range of bioactivity, such as neuroprotective, antinflammatory and immunomodulator, hepatoprotective and cardioprotective effects. Anticancer, antioxidant, antimicrobic, hypoglycaemic, hypolipidemic, choleretic, antispasmodic and purgative properties were also reported [4]. Verbascoside has been reported like a phenylenthanoid glycoside with different activities, *i.e.*, strong anti-leukaemic and cytotoxic activity against a murine cell line and anti-inflammatory activity [5]. Verbascoside also has antioxidant activity and decreases NOS activities and reduces NF-ĸβ activation and nuclear translocation and thus may modulate inflammatory reactions [6]. This study deals with the anti-inflammatory effects of ethanolic extract from *Castilleja tenuiflora* in order to evaluate the folkloric information and isolation and chemical characterization of the active constituent(s) through biossays-guided.

## 2. Results and Discussion

*Castilleja tenuiflora* is used in Mexican traditional medicine for the treatment of coughs, snake bites, and inflamed ovaries among others. Recently, a relationship between the pathophysiology of many of these diseases and the inflammatory process has been reported. Medicinal plants are important sources of numerous chemical substances with potential therapeutic effects. The use of medicinal plants for the treatment of many diseases is associated with folk medicine throughout the World. Numerous compounds have been characterized from plants. Research on plants with alleged folkloric use as pain relievers and anti-inflammatory agents has proven fruitful and is a logical research strategy in the search for new anti-inflammatory drugs. For this reason, *Castilleja tenuiflora* was chosen as a suitable candidate for the isolation of compounds with anti-inflammatory activity. 

**Figure 1:**
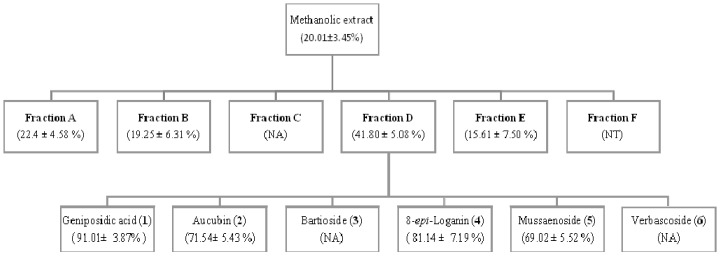
The anti-inflammatory activity of the methanolic extract and fractions of *Castilleja tenuiflora* on TPA-induced mouse ear edema. The methanolic extract was evaluated at 1 mg/ear while fractions and compounds were tested at 0.1 mg/ear. Groups of mice (*n* = 5). The data are expressed as the means of inhibition percentage (%I) ± SEM. Numbers in bold type correspond to structure number. NT: Test was performed but data could not be obtained. NA: Not analyzed.

The methanolic extract obtained from the aerial parts of *Castilleja tenuiflora* was tested in the topical model of inflammation [TPA-induced ear edema in mice (1 mg/ear)] and produced a significant effect of 20% inhibition. In contrast, the control, indomethacin, showed 40% inhibition. Six fractions (A–F) were obtained by chromatographic fractionation of the methanolic extract. Fraction D showed activity, with 42% inhibition, which was comparable to indomethacin at 1 mg/ear. The D fraction was then further analyzed by MPLC because it was the most active fraction (Figure 1).

**Figure 2:**
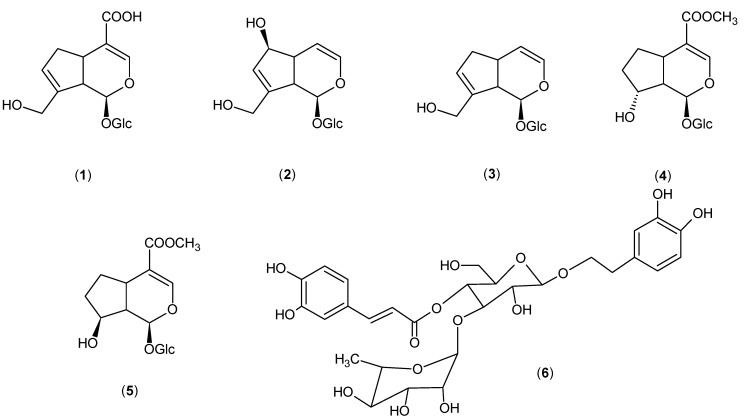
Chemical structures of the compounds isolated from *Castilleja tenuiflora.*

Fraction D was subjected to MPLC (C-column) elution with H_2_O-MeOH (10:0 to 2:1). This process yielded (in order of elution) geniposidic acid (**1**), aucubin (**2**), bartsioside (**3**), 8-*epi*-loganin (**4**), mussaenoside (**5**), and the phenylpropanoid verbascoside (**6**). The molecular structures of these compounds are shown in Figure 2. The compounds were identified by comparison of the NMR spectra with those of authentic samples.

**Figure 3:**
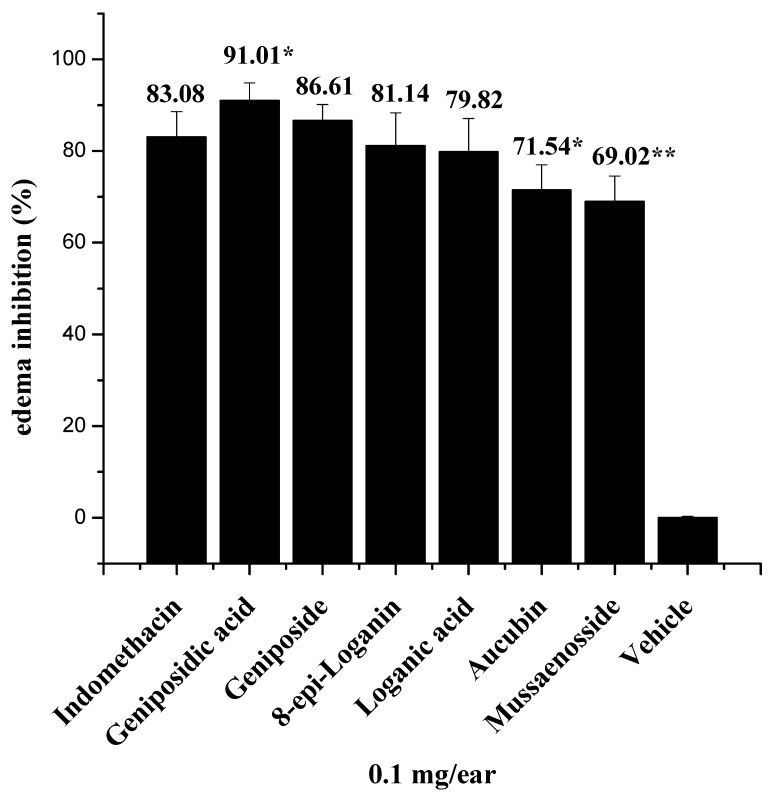
Effect of the iridoids isolated from the aerial parts of *Castilleja tenuiflora* on TPA-induced mouse ear edema. Groups of mice (*n* = 5) were treated at 0.1 mg/ear. The animals were killed four hours after the induction of inflammation. A punch biopsy was performed on each ear. The percentage of edema inhibition was calculated. The data are expressed as the means ± SEM. The differences between the control (indomethacin) and the treated groups with * *p* < 0.005 and ** *p* < 0.01 were considered significantly different compared to the control.

All of the iridoids isolated from the aerial parts of *Castilleja tenuiflora* that were tested (topical administration at 0.1 mg/ear) in the TPA-induced ear edema in mice model showed anti-inflammatory activity (Figure 3). Loganic acid (**7**), 8-*epi*-loganin (**4**), and geniposide (**8**) were not significantly different from one another, whereas aucubin (**2**), mussaenoside (**5**), and geniposidic acid (**1**) showed activity similar to that of indomethacin. Bartioside (**3**) could not be evaluated because it was isolated in very small amounts and impure.

We found that the members of the *Castilleja* family are rich in iridoids, and some of these compounds have reported anti-inflammatory activity and mechanisms of action. For example, one study evaluated the inhibition of COX-2 and COX-1 by certain iridoids, including aucubin (**2**), catalpol, gentiopicroside, swertiamarin, geniposide, geniposidic acid (**1**), and loganin. This previous study reported that the product from the hydrolysis of aucubin (H-aucubin) using β-glucosidase showed moderate inhibition of COX-2 with an IC_50_ of 8.83 mM but much less inhibition (IC_50_ 68.9 mM) of COX-1. H-Loganin and the H-geniposide exhibited higher inhibitory effects on COX-1, with IC_50_ values of 3.55 and 5.37 mM, respectively. H-Aucubin, H-catalpol H-geniposide, and H-loganin suppressed TNF-α formation with IC_50_ values of 11.2, 33.3, 58.2, and 154.6 mM, respectively. Finally, H-aucubin was shown to exhibit a significant suppression of NO production with an IC_50_ value of 14.1 mM [7].

We isolated aucubin (**2**) from *Castilleja tenuiflora* and demonstrated its anti-inflammatory activity (71.54% ± 5.43% inhibition), corroborating previously reported results. The most active iridoid was geniposidic acid (**1**) (91.01% ± 3.87% inhibition). Previous studies observed the increased anti-inflammatory activity of geniposidic acid (**1**) on carrageenan-induced hind paw edema in mice (53.3% ± 4.8% inhibition) in comparison with the control indomethacin (33.0% ± 3.1% inhibition) [8].

Geniposide (**8**) can directly bind to LPS, thus neutralizing LPS *in vitro*, which significantly protects mice from sepsis [9]. In addition, it inhibits the production of exudates and nitric oxide (NO) in the rat air pouch edema model [10]. Sepsis is known to be related to anti-inflammatory activity due to the central role of TNF-α in the chronic damage produced in organs when controlling a cascade of pro-inflammatory cytokines [11]. Similarly, the anti-inflammatory activity of loganin and loganic acid (**7**) has been demonstrated using carrageenan-induced edema and edema models instead of the TPA model in mice [12]. The remaining iridoids isolated from *Castilleja tenuiflora*, *i.e.*, 8-*epi*-loganin (**4**), and mussaenoside (**5**), had not previously been reported to have anti-inflammatory activity.

**Figure 4:**
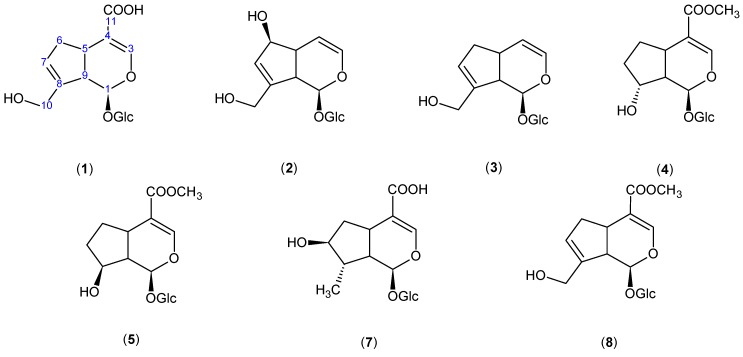
Chemical structures of the compounds evaluated on TPA-induced mouse ear edema.

We evaluated almost all of the isolated iridoids to compare their activity because previous studies have not evaluated these iridoids in the same model. In the TPA inflammatory model, the iridoids significantly inhibited ear edema. Inflammation induced by TPA can activate protein kinase C in a manner similar to that of endogenous diacylglycerol and can activate phospholipase A_2_. The activation of protein kinase C, a calcium-dependent enzyme, induces the degranulation of neutrophils, platelets, and mast cells and induces smooth muscle contraction [13]. Whereas the early phase of carrageenan edema is related to the production of histamine, leukotriene, platelet-activating factor, and cyclooxygenase products, the delayed phase has been linked to neutrophil infiltration and the production of neutrophils [14]. The compounds evaluated are showed in the Figure 4.

Verbascoside and isoverbascoside were recently isolated from the root cultures of *Castilleja tenuiflora* [1], and verbascoside (**6**) has been the subject of biological studies demonstrating anti-inflammatory activity in several models, such as the intestinal inflammation model, where it was shown to decrease the presence of pro-inflammatory molecules [15]. The ear edema test for arachidonic acid and carrageenan-induced edema showed the inhibition of the histamine and bradykinin production [16]. The inhibitory effect of the mediators of lipopolysaccharide and IFN-γ stimulated macrophages showed the potent inhibition of NO, TNF-α, and IL-12 [17] and selective inhibition of COX-2 [18,19].

In an attempt to identify the preliminary structure and activity relationship of the iridoids isolated from *Castilleja tenuiflora*, we acquired geniposide (**8**) and loganic acid (**7**) to demonstrate the influence of the electron withdrawing group in position 11 of the basic structure of an iridoid. We did not find a relationship between the structure and activity of the different iridoids evaluated. Thus, we evaluated additional compounds possessing other functional groups. We found that it is important to have an electron attractor group in position 11 (C=O), hydroxyl group in position 10 and an whole cyclopentane ring. We believe that the double bond in positions 7 and 8 is less important because the activity does not decrease significantly when the double bond is absent. We emphasize that to determine the structure-activity relationship, it is necessary to evaluate a greater number of compounds with other functional groups and to perform other tests to corroborate our preliminary analysis. Additionally, according to Liu and Wang [20] numerous studies from clinical trial as well as animal studies have shown that few toxic effects or side effects are found with treatment of iridoid glycosides.

## 3. Experimental

### 3.1. General Procedures

Preparative open column chromatography (CC) was carried out on a silica gel (70 × 5 cm I.D.; 0.063–0.200 mm, 0.2–0.5 mesh; Merck, Whitehouse Station, NJ, USA) column eluted with organic solvents or binary mixtures of solvents of increasing polarity (hexane, dichloromethane, ethyl acetate, and methanol); TLC was performed on precoated silica gel 60 F_254_ plates (Merck). Reverse phase MPLC: Merck Lobar C-18 columns size CD. H_2_0-MeOH mixtures were used as eluents and peaks were detected by UV at 210 nm.

### 3.2. Plant Material and Extraction

The aerial parts of *Castilleja tenuiflora* were collected in Zempoala Lake located in Morelos, Mexico in September 2008. Voucher specimens were deposited at the Herbarium of the University of Morelos (HUMO) deposited in the Centro de Investigación en Biodiversidad y Conservación (CIByC) of the Universidad Autónoma del Estado de Morelos, and were authenticated by Dr. J. R. Bonilla-Barbosa (Bonilla-616). Fresh whole plants (255 g) were extracted twice with absolute ethanol (0.4 L), filtered, dried, and partitioned in H_2_O-Et_2_O. The aqueous layer was concentrated, redissolved in MeOH and treated with activated charcoal. Evaporation of the filtrate gave a white foam (24.8 g), which was subjected to preparative open column chromatography (CC).

### 3.3. Chromatography Analyses

Preparative open column chromatography (CC) was carried out on a silica gel column (70 × 5 cm I.D.; 0.063–0.200 mm, 0.2–0.5 mesh; Merck) eluted with organic solvents or binary mixtures of solvents of increasing polarity (hexane, dichloromethane, ethyl acetate, and methanol). The MeOH extract (20 g) was fractionated eluting with Hex-CH_2_Cl_2_-AcOEt-MeOH mixtures of increasing polarity to yield six fractions: A, 0.1003 g, (1:1:0:0), B, 1.202 g (0:1:0:0), C, 0.7713 g (0:1:1:0), D, 7.978 g (0:0:1:0), E, 0.9947 g (0:0:1:1) and F, 4.876 g (0:0:0:1). Fractions were combined by TLC using ceric (IV) sulphate as detection. TLC was performed on precoated silica gel 60 F_254_ plates (Merck).

Fraction D (7.2 g) was separated using a reverse phase medium-pressure liquid chromatography (MPLC) column with a CD size (packed with Merck Lobar C-18) of 800 mm in length and 47 mm in diameter. The chromatography was performed in gradient mode using a flow rate of 24 mL/min. The solvents were HPLC grade water and methanol. The detection was conducted at 206 and 240 nm using a UV detector (Shimadzu SPD-10A). This separation yielded (in order of elution): geniposidic acid (H_2_O:MeOH 10:0; 0.208 g; 0.32%; t_R_ 1.4 min), aucubin (H_2_O:MeOH 15:1; 0.86 g; 1.32%; t_R_ 1.7 min), bartsioside (H_2_O:MeOH 3:1; 0.089 g; 0.14%; t_R_ 6.0 min), 8-*epi*-loganin (H_2_O:MeOH 3:1; 0.057 g; 0.09%; t_R_ 6.7 min), mussaenoside (H_2_O:MeOH 3:1; 0.13 g; 0.19%; t_R_ 6.9 min), and verbascoside (H_2_O:MeOH 1:1; 0.46 g; 0.71%; t_R_ 7.4 min).

### 3.4. Spectroscopic Data

^1^H, ^13^C-NMR and 2D spectra were recorded using Varian 300 and 500 MHz instruments. The samples were dissolved in deuterated methanol (CD_3_OD) or deuterated water (D_2_O). In the NMR spectra, the solvent peaks were used as the internal standard. The isolated compounds were identified by comparison with NMR spectra of the reference samples and/or published NMR data.

### 3.5. Chemicals

TPA and indomethacin were purchased from Sigma-Aldrich Chemical Co. (St. Louis, MO, USA), geniposide was purchased from ChromaDex^®^ (Irvine, CA, USA) and loganic acid was donated by S. R. Jensen, Technical University of Denmark. All other chemicals and reagents were of the highest commercial grade available.

### 3.6. Anti-Inflammatory Assay

Male ICR mice, weighing 25–30 g each, were used. The Instituto de Biotecnología, Universidad Nacional Autónoma de México, provided the experimental animals. All animals were kept under standard laboratory conditions (temperature 27 ± 1 °C). They had access to pelleted food and water *ad libitum*. The animals were assigned to different treatment groups (5 per group). The animal studies were conducted in accordance with the international guidelines for the use and care of animals.

For testing edema induced with acetate 12-O-tetradecanoylphorbol (TPA), the mouse model of acute inflammation used in these studies was a slight modification of a previously described procedure [21]. The right ear of each ICR mouse (five per group) was topically treated with sample (0.1 mg or 1 mg/ear) in 20 µL of vehicle (acetone) 30 min prior to the application of 2.5 µg/ear TPA in 20 µL of acetone. The left ears were treated with vehicle alone, and the control mice received only vehicle on both ears. Four hours later, the mice were killed by cervical dislocation. An 8 mm diameter plug was removed from each ear. The swelling was assessed as the difference in weight between the right and left ear plugs. The data are expressed as the standard error of the mean (SEM) of 5 mice. Inhibition of edema (EI, %) was calculated using the following equation:

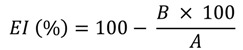
(1)
where A = edema induced by TPA alone, and B = edema induced by TPA plus sample. The extract and fractions were tested at doses of 1 mg/ear, whereas all pure compounds were tested at 0.1 mg/ear.

### 3.7. Data Analysis

The data analysis was performed using ORIGIN^®^ version 8.0. All data are expressed as the means ± SEM, and statistical significance was determined via Student’s t-test with *p* < 0.05 considered to be significant.

## 4. Conclusions

The results of these investigations confirm the great potential of Mexican medicinal plants for the production of bioactive compounds and validate the ethnomedical use of *Castilleja tenuiflora-*like anti-inflammatory plants. The anti-inflammatory activity of 8-*epi*-loganin (**4**) and mussaenoside (**5)** has not been previously reported. It is possible that the structure necessary for the anti-inflammatory activity of iridoids is the basic structure of an iridoid with an electron withdrawing group in position 11 (C=O) and a hydroxyl group in position 10; however, additional tests are necessary to confirm this theory.
